# Epigallocatechin-3-Gallate Protects Trabecular Meshwork Cells from Endoplasmic Reticulum Stress

**DOI:** 10.1155/2022/7435754

**Published:** 2022-11-10

**Authors:** Linbin Zhou, Jing Na He, Lin Du, Bo Man Ho, Danny Siu-Chun Ng, Poemen P. Chan, Clement C. Tham, Chi Pui Pang, Wai Kit Chu

**Affiliations:** ^1^Department of Ophthalmology & Visual Sciences, The Chinese University of Hong Kong, Hong Kong; ^2^Lam Kin Chung. Jet King-Shing Ho Glaucoma Treatment and Research Centre, The Chinese University of Hong Kong, Hong Kong

## Abstract

Primary open-angle glaucoma (POAG) is the most common form of glaucoma, for which elevated intraocular pressure (IOP) is a major risk factor. IOP is mainly regulated by dynamic balance of aqueous humor (AH) production and outflow via the conventional trabecular meshwork/Schlemm's canal (TM/SC) pathway. Dysfunctions of TM cells due to endoplasmic reticulum (ER) stress have been demonstrated to increase the resistance of AH outflow, resulting in IOP elevation. Epigallocatechin-3-gallate (EGCG), the most abundant polyphenolic component in green tea, has been shown to alleviate ER stress in several diseases while its potential roles in alleviating ER stress in TM cells have not been determined. In this study, we investigate the mitigation of tunicamycin-induced ER stress in TM cells by EGCG. MTT assay was used to measure the cell viability of human TM (HTM) cells and primary porcine TM (PTM) cells. ER stress levels in both HTM cells and primary PTM cells were detected by quantitative real-time PCR. The primary PTM cells isolated from porcine TM tissues were characterized by immunostaining. We found that 40 *μ*M and 80 *μ*M EGCG pretreatment substantially promoted HTM cell survival under 3 *μ*M tunicamycin-induced ER stress. Pretreatment of 40 *μ*M EGCG markedly reduced the expression of ER stress markers *ATF4*, *HSPA5*, and *DDIT3,* evoked by 3 *μ*M tunicamycin in HTM cells. Furthermore, 40 *μ*M EGCG pretreatment significantly decreased the expressions of *ATF4*, *HSPA5*, and *DDIT3* at the mRNA level induced by 3 *μ*M tunicamycin and improved cell viability in primary PTM cells. Our results show that EGCG is capable of protecting TM cells from ER stress. EGCG provides a promising therapeutic option for POAG treatment.

## 1. Introduction

Glaucoma, a leading cause of irreversible blindness worldwide, is a heterogenous group of optic neuropathies characterized by degeneration of the optic nerve, progressive loss of retinal ganglion cells, and impaired visual field [1–3]. It is estimated that the number of people with glaucoma would reach 111.8 million in 2040 [4]. Primary open-angle glaucoma (POAG) is the most common form of glaucoma, and elevated intraocular pressure (IOP) is one of the major risk factors for POAG [5]. Thus far, lowering IOP via medication and glaucoma filtration surgeries are proven efficacious intervention approach to delaying the progression of the disease [5].

IOP is regulated by dynamic balance of aqueous humor (AH) production and outflow [6]. The pathways of AH outflow include the conventional trabecular meshwork/Schlemm's canal (TM/SC) pathway and the unconventional uveoscleral pathway [7]. The conventional TM/SC outflow pathway is a key element in regulating AH drainage, where the cribriform meshwork layer and the inner wall of endothelial cells of the SC form the main outflow resistance [8]. Abnormalities in the trabecular meshwork cells in the TM/SC pathway due to intrinsic or extrinsic factors, including gene mutations [9], oxidative stress [10], and glucocorticoid administration [11] can lead to increased AH drainage resistance and elevated IOP.

Endoplasmic reticulum (ER) stress is associated with TM dysfunction and the development of POAG [12–14]. ER is engaged in the synthesis and the processing of secretory and membrane proteins [15]. Properly folded proteins are transported to the Golgi apparatus, while misfolded proteins are removed by ER-associated degradation (ERAD) in the ER [16]. When the amount of misfolded proteins exceeds the degradation capacity of the ERAD machinery, the misfolded proteins tend to accumulate in the ER and lead to ER stress [15]. To alleviate such stress in eukaryotic cells, the unfolded protein response (UPR) is activated to restore the ER homeostasis [15]. Activation of the UPR involves ER stress sensing through inositol-requiring enzyme 1 (IRE1), RNA-dependent protein kinase (PKR)-like ER kinase (PERK), and activating transcription factor 6 (ATF6), which then finely regulates the response via inducing ER chaperones heat shock protein A5 (HSPA5) and glucose-regulated protein 94 (GRP94), activation of activating transcription factor 4 (ATF4), eukaryotic initiation factor 2 alpha (eIF2*α*) phosphorylation, and alternative splicing of X-box binding protein 1 (XBP1) [15, 17, 18]. When ER stress persists and the UPR adaptive response fails to resolve the overwhelming loading of misfolded proteins, the ER stress can give rise to cell death via induction of DNA damage induced transcript 3 (DDIT3), ER-specific caspase 12, and several other factors [19–21]. The unmitigated ER stress, resulting from gene mutations, oxidative stress, and glucocorticoid administration, can cause dysfunction and even death of the TM cells, leading to increased resistance to AH drainage and IOP elevation [22–24].

Epigallocatechin-3-gallate (EGCG), the most abundant polyphenolic constituent of green tea, possesses potent antioxidant, anti-inflammatory and antiapoptotic properties [25–27]. It has been reported that EGCG could suppress ER stress mediated-apoptosis to protect mice against cisplatin-induced nephrotoxicity [28]. Additionally, EGCG has been demonstrated to alleviate ER stress to exert beneficial effects on pathological conditions such as amyloid beta-induced neurotoxicity [29] and high glucose-induced apoptosis in podocytes [30]. It has also been reported that EGCG could promote autophagy-dependent survival through finely regulating the balance of the mTOR-AMPK pathway upon ER stress challenge [31]. Nonetheless, it remains to be determined if EGCG can suppress ER stress and enhance TM cell survival. In the current study, we aimed to investigate the potential roles of EGCG in protecting TM cells from ER stress.

## 2. Materials and Methods

### 2.1. Cell Culture and Reagents

Human trabecular meshwork (HTM) cell line was isolated by Polansky et al. [32]. The primary cells were transfected with a SV40 origin defective vector by Filla et al. to established the immortalized HTM cell line [33]. Primary porcine trabecular meshwork (PTM) cells were isolated from trabecular meshwork tissues of fresh porcine eyes. To establish the primary trabecular meshwork cells, we followed the consensus recommendations to combine TM cells isolated from 12 porcine eyes, which could minimize the biological variations in our experimental data due to genetic and sex variations from a single source animal [34]. Briefly, 12 fresh porcine eyes were immersed in 70% ethanol for 2 min. The eyeballs were then rinsed three times with sterile phosphate buffer saline (PBS). Under microscope, a cross incision was made at the posterior end of the eyeball followed by extension of the incision to the regions 1-2 mm posterior to the limbus. The vitreous body and the retina were removed by inverse lifting the eyeball with cornea facing upward. A 360-degree incision was further made 1-2 mm posterior to the limbus to remove the redundant sclera. The ciliary body and the iris were removed by forceps without damaging the angle regions. Using fine-tooth forceps and under microscopic high-power magnification, a light-gray line of tissue was isolated from the area just anterior to the limbus. Then, the light-gray explants were cut into small pieces and placed into a culture dish. The explants were cultured in Dulbecco's modified Eagle's medium (DMEM) (Gibco, Cat# 31600034) supplemented with 10% fetal bovine serum (FBS) and containing antibiotics (100 U/mL penicillin and 100 *μ*g/mL streptomycin) in a 37°C humidified incubator with 5% CO_2_ and filtered air for 1 week until the tissues had adhered to the dish. The medium was changed every 2-3 days. Primary PTM cells before passage 6 were used for the experiments. Both HTM and primary PTM cells were maintained in DMEM medium supplemented with 10% FBS and containing antibiotics (100 U/mL penicillin and 100 *μ*g/mL streptomycin) in a 5% CO_2_ humidified incubator at 37°C.

Tunicamycin (Sigma Aldrich, Cat# 11089-65-9) and dexamethasone (Sigma Aldrich, Cat# D4902) were dissolved in dimethyl sulfoxide (DMSO) to make a 3 mM and 2 mM stock solution, respectively, while EGCG (Sigma Aldrich, Cat# E-4143) was dissolved in sterile double distilled water and filtered to make a 20 mM stock solution. All stock solutions were stored in a -20°C freezer.

### 2.2. MTT Assay

Viability of the cells was determined by MTT assay [35]. Briefly, 8 × 10^3^ HTM cells or primary PTM cells were seeded per well in 96-well plate, and at least triplicate wells were designated for each treatment group. EGCG and/or tunicamycin of indicated concentrations were treated to HTM cells and primary PTM cells for indicated periods of time. Then, 10 *μ*L MTT (10 *μ*g/mL) (Thermo Fisher, Cat# M6494) was directly added to each well and incubated with the cells at 37°C for 4 h. The media were then discarded completely, and 150 *μ*L DMSO was added to each well to dissolve the crystals for 10 min. Absorbance was detected at 570 nm by a microplate reader (PowerWave™ XS Microplate Reader, BioTek, VT).

### 2.3. Quantitative Real-Time Polymerase Chain Reaction (PCR)

Cells were lysed in Trizol (Thermo Fisher, Cat# 15596026) on ice for 5 min. Total RNA was extracted and treated with RNase-free DNase I (Qiagen, Cat# 79256) according to the manufacturer's instructions. 1.5 *μ*g RNA of each sample was reverse transcribed into cDNA by using SuperScript III reverse transcriptase (Thermo Fisher, Cat# 18080044) in an iCycler PCR instrument (Bio-Rad). The cDNA products were further used to measure the gene expression by a LightCycler 480 II real-time PCR instrument (Roche Applied Science). Gene expression was calculated using the 2^−ΔΔCt^ method, and *ACTB* transcription levels were utilized as an internal control [36]. Sequences of specific primers for each detected gene are listed in Table 1.

### 2.4. Immunostaining

Primary PTM cells were seeded on coverslips in a 6-well plate overnight. 1 *μ*M dexamethasone (Sigma Aldrich, Cat# D4902) or DMSO in culture medium was used to treat primary PTM cells for 48 h. Cells on the coverslips were then rinsed with PBS twice and fixed with 4% paraformaldehyde for 15 min at RT, which were followed by permeabilization with 0.5% TritonX-100 and blocking with 3% bovine serum albumin (BSA) for 1 h at RT. Then, the cells were incubated with myocilin (1 : 100, Imgenex, self-made) primary antibody at 4°C overnight. Cells were incubated with a secondary antibody (1 : 500) for 1 h at RT. After incubation, cells were stained with DAPI for 5 min, washed with PBST thrice (10 min for each time) at RT to remove unbounded antibody, and then were proceeded to detection under a fluorescence microscope (A1MP, Nikon).

### 2.5. Statistical Analysis

All data were presented as mean ± standard deviation (SD). GraphPad Prism 5.0 software was used for statistical analyses. Unpaired Student's *t*-test was used to compare the differences between two groups, while one-way ANOVA test was utilized to compare the differences among multiple groups. Differences were considered statistically significant when *p* < 0.05.

## 3. Results

**Figure 1:**
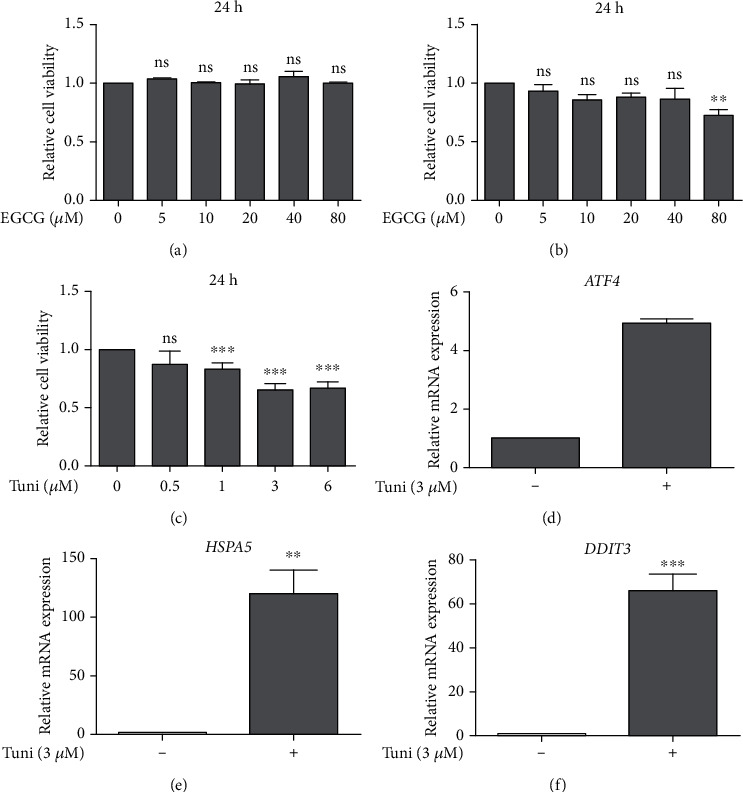
Cell viability of HTM cells was measured by MTT assay after various doses (0, 5, 10, 20, 40 and 80 *μ*M) of EGCG treatment for 24 h (a) (*n* = 4) and 48 h (b) (*n* = 4). Cell viability of HTM cells was determined via MTT assay after various doses (0, 0.5, 1, 3 and 6 *μ*M) of tunicamycin (Tuni) treatment for 24 h (c) (*n* = 4). Changes in the relative mRNA expression of ER stress markers including *ATF4* (d) (*n* = 4), *HSPA5* (e) (*n* = 4), and *DDIT3* (f) (*n* = 4) in HTM cells after 3 *μ*M Tuni treatment for 24 h were detected through quantitative real-time PCR assay. Data shown are mean ± standard deviation of representative experiments. (a, b) Comparisons were performed in treatment groups versus 0 *μ*M EGCG group. (c–f) Comparisons were performed in treatment groups versus 0 *μ*M Tuni group; ns: not significant. ^∗∗^*p* < 0.01, ^∗∗∗^*p* < 0.001.

To determine if EGCG could have a protective role against ER stress in the TM cells, we first tested the effects of EGCG on the viability of HTM cells. EGCG of various dosages ranging from 10 *μ*M to 80 *μ*M was used to treat HTM cells for 24 h and 48 h, and the effects of different dosages of EGCG on the viability of HTM cells were quantified by MTT assay. As shown in Figures 1(a) and 1(b), treating HTM cells with a high concentration of EGCG (80 *μ*M) for 24 h did not affect HTM cell viability. However, with prolonged treatment for 48 h, EGCG at high dosage (80 *μ*M) significantly reduced HTM cell viability. Treatment with EGCG at low dosages (10 *μ*M, 20 *μ*M, and 40 *μ*M) for 48 h also slightly decreased HTM cell survival, although not statistically significant. The effects of tunicamycin, an ER stress inducer which functions via inhibiting protein glycosylation to potently induce ER stress [37], on HTM cell survival were also detected. Treatment with 1 *μ*M, 3 *μ*M, and 6 *μ*M tunicamycin for 24 h significantly reduced HTM cell viability, while the detrimental effects of 3 *μ*M and 6 *μ*M tunicamycin were similar and more severe than that of 1 *μ*M tunicamycin (Figure 1(c). Furthermore, 3 *μ*M tunicamycin induced strong ER stress in HTM cells as revealed by markedly enhanced expression of ER stress markers *ATF4*, *HSPA5*, and *DDIT3* (Figures 1(d)–1(f)), which are mediators with important functions in ER stress (Table 2). Therefore, 3 *μ*M tunicamycin was selected to establish the ER stress cellular model in HTM cells to test the protective roles of EGCG against ER stress in TM cells.

**Figure 2:**
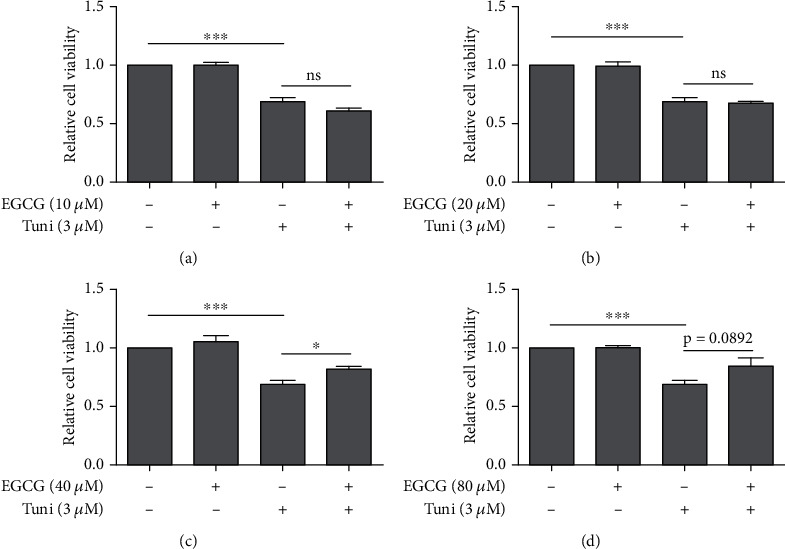
EGCG pretreatment inhibited Tuni-induced decrease in cell viability of HTM cells. HTM cells were pretreated with various doses (10, 20, 40, and 80 *μ*M) of EGCG for 24 h, followed by 3 *μ*M Tuni treatment for 24 h. Cell viability of HTM cells was measured by MTT assay to determine the pretreatment effects of 10 *μ*M (a) (*n* = 4), 20 *μ*M (b) (*n* = 4), 40 *μ*M (c) (*n* = 4), and 80 *μ*M (d) (*n* = 4) EGCG on the viability of 3 *μ*M Tuni-treated HTM cells. Data shown are mean ± standard deviation of representative experiments; ns: not significant. ^∗^*p* < 0.05, ^∗∗∗^*p* < 0.001.

Next, we investigated if EGCG could protect HTM cells from tunicamycin-induced ER stress. HTM cells were pretreated with EGCG at various dosages for 24 h, followed by 3 *μ*M tunicamycin treatment for another 24 h in the absence of EGCG. Cell viability assay results revealed that 10 *μ*M and 20 *μ*M EGCG failed to improve HTM cell survival under tunicamycin-induced ER stress (Figures 2(a) and 2(b)). Nonetheless, 40 *μ*M EGCG partially rescued HTM cells from ER stress with significantly improved cell viability in comparison with 3 *μ*M tunicamycin treatment alone group (Figure 2(c)). In addition, 80 *μ*M EGCG also partially promoted HTM cell survival under tunicamycin-induced ER stress although the statistical analysis was not significant (Figure 2(d)). These results suggest that EGCG improves HTM cell viability under tunicamycin-induced ER stress.

**Figure 3:**
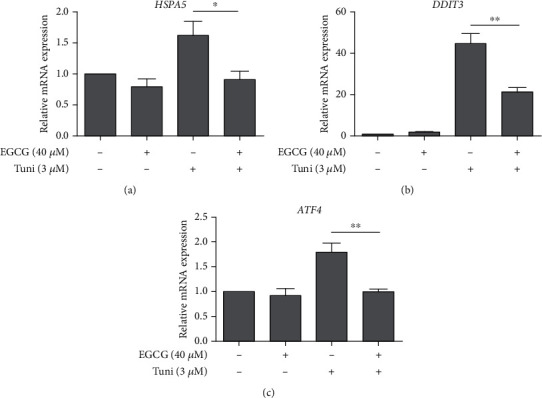
EGCG pretreatment mitigates Tuni-induced ER stress in HTM cells. HTM cells were pretreated with 40 *μ*M EGCG for 24 h, followed by 3 *μ*M Tuni treatment for 6 h (a–c). Changes at the mRNA levels of ER stress markers including *HSPA5* (a) (*n* = 4), *DDIT3* (b) (*n* = 4), and *ATF4* (c) (*n* = 4) in HTM cells were detected by quantitative real-time PCR assay. Data shown are mean ± standard deviation of representative experiments; ns: not significant. ^∗^*p* < 0.05, ^∗∗^*p* < 0.01.

To further confirm EGCG could alleviate ER stress to promote HTM cell viability under tunicamycin-induced ER stress, expression of ER stress markers including *HSPA5*, *DDIT3*, and *ATF4* in HTM cells were measured at the mRNA level. As shown in Figures 3(a)–3(c), 3 *μ*M tunicamycin treatment alone induced strong expressions of *HSPA5*, *DDIT3*, and *ATF4* in HTM cells at the mRNA levels, whereas 40 *μ*M EGCG pretreatment notably suppressed their expression. These results indicate that EGCG can promote HTM cell viability by inhibiting ER stress.

**Figure 4:**
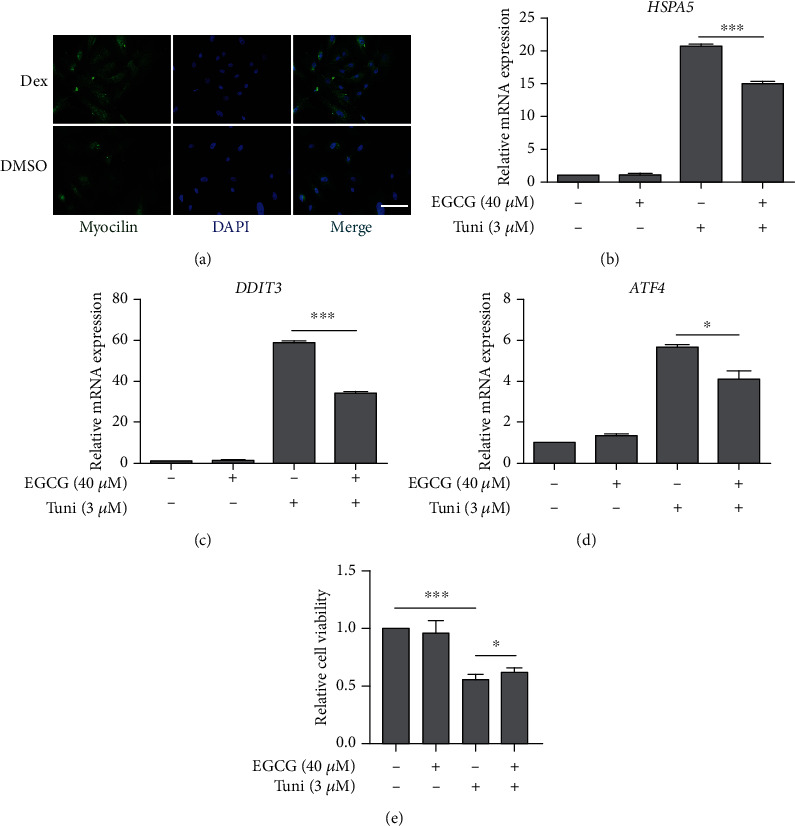
EGCG pretreatment alleviates Tuni-induced ER stress and enhances cell viability in primary PTM cells. (a) Primary PTM cells isolated from porcine TM tissues were treated with 1 *μ*M dexamethasone (Dex) or DMSO for 48 h, and the induction of myocilin expression was evaluated by immunostaining. Primary PTM cells were pretreated with 40 *μ*M EGCG for 24 h, followed by 3 *μ*M Tuni treatment for 6 h (b–d) and for 24 h (e). Changes at the mRNA levels of ER stress markers including *HSPA5* (b) (*n* = 4), *DDIT3* (c) (*n* = 4), and *ATF4* (d) (*n* = 4) in primary PTM cells were determined by quantitative real-time PCR assay. Cell viability of primary PTM cells was measured by MTT assay to determine the pretreatment effects of 40 *μ*M EGCG on the viability of 3 *μ*M Tuni-treated primary PTM cells (e) (*n* = 4). Data shown are mean ± standard deviation of representative experiments. Scale bar, 100 *μ*m. ^∗^*p* < 0.05, ^∗∗∗^*p* < 0.001.

Immortalized human TM cell lines could lose some of the properties of nonimmortalized TM cells, and thus findings in immortalized human TM cell lines must be replicated in nonimmortalized TM cells [34]. We further validated our results by using primary TM cells isolated from porcine TM tissues. As shown in Figure 4(a), treatment with 1 *μ*M dexamethasone for two days notably enhanced the expression of myocilin in these primary porcine cells, confirming their identity as primary porcine TM (PTM) cells. We then tested if EGCG could reduce tunicamycin-induced ER stress and improve cell viability in these primary PTM cells. Pretreatment by 40 *μ*M EGCG significantly inhibited the expression of the ER stress markers *HSPA5*, *DDIT3*, and *ATF4* at the mRNA levels (Figures 4(b)–4(d)) and notably improved primary PTM cell viability (Figure 4(e)) compared to the 3 *μ*M tunicamycin treatment alone group. Taken together, these results indicate the protection of TM cells under ER stress by EGCG.

## 4. Discussion

Dysfunction or loss of the TM cells is widely thought to be a major cause of glaucoma [38]. Accumulative ER stress in the TM cells due to gene mutations, oxidative stress, and glucocorticoid administration exerts detrimental effects to the functions and viability of TM cells, which contribute to the development and progression of the disease [10, 22–24]. Experimental evidence indicates that EGCG can improve cell viability via inhibition of ER stress in multiple pathological conditions [28–31]. The protective effects of EGCG on TM cells under ER stress had not been clarified. In this study, we demonstrate that EGCG can suppress ER stress and promote TM cell viability. We show that EGCG pretreatment promoted cell viability under tunicamycin-induced ER stress and reduced the ER stress levels in both HTM cells and primary PTM cells.

EGCG is the most active and abundant constituent of green tea [39]. Multiple studies have demonstrated that EGCG is able to promote cell survival by suppressing ER stress under varieties of pathological conditions [28–30]. Nonetheless, EGCG has also been reported to trigger ER stress and apoptosis in tumor cells [40, 41]. In this study, treatment by low dose of EGCG (40 *μ*M) for 24 h did not affect HTM cell viability (Figure 1(a)) but protect HTM cells from tunicamycin-induced ER stress (Figure 2(c) and Figures 3(a)–3(c)). High-dose EGCG (80 *μ*M) treatment for 48 h but not 24 h substantially decreased HTM cell viability (Figure 1(b)), which indicated the cellular cytotoxicity of high-dose EGCG treatment in HTM cells. Our observations are similar to the results of the investigation in HEK293T cells, in which 80 *μ*M EGCG treatment for 24 h increased ER stress and reduced the cell viability by around 50% [31]. Moreover, in colorectal cancer cells, a very high dose (125 *μ*M) of EGCG dramatically reduced cell viability via ER stress induction [41]. Although a very low concentration (1-2 *μ*M and up to 10 *μ*M) of EGCG could generate low level of intracellular reactive oxygen species (ROS) which stimulated transduction of multiple signals to enhance cellular protective mechanisms [42, 43], high concentrations (>50 *μ*M) of EGCG could exert strong prooxidant actions to decrease cell viability via induction of ROS in several types of cancer cells [44–46]. Furthermore, as oxidative stress could result in ER stress and activation of ER stress-related apoptosis pathway [47], high-dose EGCG might impair cell survival via ROS-induced ER stress. In addition to ER stress induction, high concentrations of EGCG can also decrease cell viability via a variety of mechanisms across different cancer cells, such as inhibiting fatty acid synthase (FASN) activity and epidermal growth factor receptor (EGFR) signaling to induce apoptosis in human adenocarcinoma lung cancer cells [48], inducing cell cycle arrest and impeding EGFR signaling to evoke cell cycle arrest and apoptosis in human epidermoid carcinoma cells [49], and inactivating *β*-catenin signaling to increase cell death of human skin cancer cells [50]. Notably, prolonged treatment (72 h) even with a low dose (40 *μ*M) of EGCG could also substantially decrease the cell viability of human adrenal cancer cells [51]. Therefore, the detrimental effects of EGCG treatment (80 *μ*M for 48 h) on HTM cell viability in our study may be attributable to these factors. These studies in conjunction with our observations suggest that the dosage and exposure time critically influence the effects of EGCG on TM cells.

Accumulating evidence has demonstrated that ER stress is upregulated in glaucomatous TM tissues of glaucoma patients and ER stress in TM tissues contributes to the development and progression of glaucoma [12, 24, 52]. ER stress arising from gene mutations, oxidative stress, and glucocorticoid administration can damage functions and survival of TM cells, resulting in increased resistance to AH outflow and elevated IOP [22–24]. In this study, EGCG pretreatment protected TM cells from tunicamycin-induced cell death via inhibiting the ER stress. EGCG could alleviate ER stress via promotion of autophagy *in vitro* [31, 53]. The underlying mechanisms accounting for the protective effects of EGCG on TM cells might be induction of autophagy in TM cells by EGCG and subsequent promotion of degradation of accumulative misfolded intracellular proteins. On the other hand, the cross-talk between oxidative stress and ER stress could contribute to dysfunction and decreased viability of TM cells [23]. Hence, as a potent antioxidant, EGCG may activate protective mechanisms [54] against oxidative stress injury to suppress the ER stress and thereby promote survival of TM cells.

Evidence from several *in vivo* studies has demonstrated the protective effects of EGCG in glaucoma models. For example, in an acute glaucoma model induced by optic nerve injury, EGCG administration alleviated optic nerve injury in the course of glaucoma through regulating the nuclear factor-*κ*B signaling pathway [55]. Additionally, in a chronic glaucoma model evoked by anterior chamber microbeads injection, EGCG protected retinal ganglion cells (RGCs) from degeneration [56]. Nevertheless, the effects of EGCG on TM cells have not been investigated in these studies. Results of the current *in vitro* study of the protective effects of EGCG on TM cells show that EGCG can inhibit ER stress to promote TM cell viability. Accordingly, EGCG should be able to not only directly protect RGCs but also protect TM cells from ER stress to indirectly reduce damage to RGCs, thus impeding glaucoma development and progression. Future investigations are warranted to address this possibility *in vivo* using glaucoma models.

## 5. Conclusions

In summary, our study shows that EGCG can reduce ER stress in TM cells and promote TM cell survival under ER stress. Administering EGCG is thus a novel and promising approach to mitigating ER stress in TM cells and potentially improving TM cell survival in POAG.

## Figures and Tables

**Table 1:** Sequences of specific primers used in this study.

Species	Gene name	Direction	Sequence (5′---3′)
Human	*HSPA5*	Forward	CTTGCCGTTCAAGGTGGTTG
		Reverse	CCTGACATCTTTGCCCGTCT
	*DDIT3*	Forward	TTCACCACTCTTGACCCTGC
		Reverse	TTCCTGCTTGAGCCGTTCAT
	*ATF4*	Forward	TCAGTCCCTCCAACAACAGC
		Reverse	CCAACGTGGTCAGAAGGTCA
	*ACTB*	Forward	GAGAAAATCTGGCACCACACC
		Reverse	GGATAGCACAGCCTGGATAGCAA

Porcine	*HSPA5*	Forward	GGTGTCTTTGAAGTCGTGGC
		Reverse	GCCCGTTTGGCCTTTTCTAC
	*DDIT3*	Forward	CCACACTCGCCTGATTCCAG
		Reverse	TGCTTGAGCCGTTCGTTCTC
	*ATF4*	Forward	AGGTTGCCCCCTTTACGTTC
		Reverse	GGGCTCATACAGATGCCACT
	*ACTB*	Forward	AGCAAGAGAGGCATCCTGAC
		Reverse	GGGGTGTTGAAGGTCTCGAA

**Table 2:** ER stress markers used in this study and their functions in ER stress.

ER stress markers	Main functions in ER stress	References
HSPA5	Initiating unfolded protein response and reduce unfolded/misfolded protein load	[57]
ATF4	Promoting aberrant protein synthesis and ER client protein load to trigger cell death	[52]
DDIT3	Inhibiting the expression of antiapoptotic factor BCL2 to hasten cell death	[19, 58]

## Data Availability

The datasets supporting the conclusions of this article are included within the article.

## References

[1] Quigley H. A., Broman A. T. (2006). The number of people with glaucoma worldwide in 2010 and 2020. *The British Journal of Ophthalmology*.

[2] Kwon Y. H., Fingert J. H., Kuehn M. H., Alward W. L. M. (2009). Primary open-angle glaucoma. *The New England Journal of Medicine*.

[3] Quigley H. A. (1999). Neuronal death in glaucoma. *Progress in Retinal and Eye Research*.

[4] Tham Y. C., Li X., Wong T. Y., Quigley H. A., Aung T., Cheng C. Y. (2014). Global prevalence of glaucoma and projections of glaucoma burden through 2040: a systematic review and meta-analysis. *Ophthalmology*.

[5] Jonas J. B., Aung T., Bourne R. R., Bron A. M., Ritch R., Panda-Jonas S. (2017). Glaucoma. *Lancet*.

[6] McDougal D. H., Gamlin P. D. (2015). Autonomic control of the eye. *Comprehensive Physiology*.

[7] Costagliola C., dell’Omo R., Agnifili L. (2020). How many aqueous humor outflow pathways are there?. *Survey of Ophthalmology*.

[8] Llobet A., Gasull X., Gual A. (2003). Understanding trabecular meshwork physiology: a key to the control of intraocular pressure?. *News in Physiological Sciences*.

[9] Vranka J. A., Kelley M. J., Acott T. S., Keller K. E. (2015). Extracellular matrix in the trabecular meshwork: intraocular pressure regulation and dysregulation in glaucoma. *Experimental Eye Research*.

[10] Saccà S. C., Gandolfi S., Bagnis A. (2016). From DNA damage to functional changes of the trabecular meshwork in aging and glaucoma. *Ageing Research Reviews*.

[11] Raghunathan V. K., Morgan J. T., Park S. A. (2015). Dexamethasone stiffens trabecular meshwork, trabecular meshwork cells, and matrix. *Investigative Ophthalmology & Visual Science*.

[12] Peters J. C., Bhattacharya S., Clark A. F., Zode G. S. (2015). Increased endoplasmic reticulum stress in human glaucomatous trabecular meshwork cells and tissues. *Investigative Ophthalmology & Visual Science*.

[13] Zode G. S., Kuehn M. H., Nishimura D. Y. (2011). Reduction of ER stress via a chemical chaperone prevents disease phenotypes in a mouse model of primary open angle glaucoma. *The Journal of Clinical Investigation*.

[14] Zode G. S., Bugge K. E., Mohan K. (2012). Topical ocular sodium 4-phenylbutyrate rescues glaucoma in a myocilin mouse model of primary open-angle glaucoma. *Investigative Ophthalmology & Visual Science*.

[15] Oakes S. A., Papa F. R. (2015). The role of endoplasmic reticulum stress in human pathology. *Annual Review of Pathology*.

[16] Smith M. H., Ploegh H. L., Weissman J. S. (2011). Road to ruin: targeting proteins for degradation in the endoplasmic reticulum. *Science*.

[17] Frakes A. E., Dillin A. (2017). The UPR^ER^: sensor and coordinator of organismal homeostasis. *Molecular Cell*.

[18] Zhu G. Y., Lee A. S. (2015). Role of the unfolded protein response, GRP78 and GRP94 in organ homeostasis. *Journal of Cellular Physiology*.

[19] Marciniak S. J., Yun C. Y., Oyadomari S. (2004). CHOP induces death by promoting protein synthesis and oxidation in the stressed endoplasmic reticulum. *Genes & Development*.

[20] Szegezdi E., Fitzgerald U., Samali A. (2003). Caspase-12 and ER-stress-mediated apoptosis. *Annals of the New York Academy of Sciences*.

[21] Hetz C. (2012). The unfolded protein response: controlling cell fate decisions under ER stress and beyond. *Nature Reviews Molecular Cell Biology*.

[22] Joe M. K., Sohn S., Hur W., Moon Y., Choi Y. R., Kee C. (2003). Accumulation of mutant myocilins in ER leads to ER stress and potential cytotoxicity in human trabecular meshwork cells. *Biochemical and Biophysical Research Communications*.

[23] Ying Y., Xue R., Yang Y. (2021). Activation of ATF4 triggers trabecular meshwork cell dysfunction and apoptosis in POAG. *Aging*.

[24] Zode G. S., Sharma A. B., Lin X. (2014). Ocular-specific ER stress reduction rescues glaucoma in murine glucocorticoid-induced glaucoma. *The Journal of Clinical Investigation*.

[25] Xie L. W., Cai S., Zhao T. S., Li M., Tian Y. (2020). Green tea derivative (−)-epigallocatechin-3-gallate (EGCG) confers protection against ionizing radiation-induced intestinal epithelial cell death both *in vitro* and *in vivo*. *Free Radical Biology & Medicine*.

[26] Li J., du L., He J. N. (2021). Anti-inflammatory effects of GTE in eye diseases. *Frontiers in Nutrition*.

[27] El-Missiry M. A., Othman A. I., El-Sawy M. R., Lebede M. F. (2018). Neuroprotective effect of epigallocatechin-3-gallate (EGCG) on radiation-induced damage and apoptosis in the rat hippocampus. *International Journal of Radiation Biology*.

[28] Chen B., Liu G., Zou P. (2015). Epigallocatechin-3-gallate protects against cisplatin-induced nephrotoxicity by inhibiting endoplasmic reticulum stress-induced apoptosis. *Experimental Biology and Medicine*.

[29] Du K., Liu M., Zhong X. (2018). Epigallocatechin gallate reduces amyloid *β*-induced neurotoxicity via inhibiting endoplasmic reticulum stress-mediated apoptosis. *Molecular Nutrition & Food Research*.

[30] Xiang C., Xiao X., Jiang B. (2017). Epigallocatechin-3-gallate protects from high glucose induced podocyte apoptosis via suppressing endoplasmic reticulum stress. *Molecular Medicine Reports*.

[31] Holczer M., Besze B., Zámbó V., Csala M., Bánhegyi G., Kapuy O. (2018). Epigallocatechin-3-Gallate (EGCG) promotes autophagy-dependent survival via influencing the balance of mTOR-AMPK pathways upon endoplasmic reticulum stress. *Oxidative Medicine and Cellular Longevity*.

[32] Polansky J. R., Weinreb R. N., Baxter J. D., Alvarado J. (1979). Human trabecular cells. I. Establishment in tissue culture and growth characteristics. *Investigative Ophthalmology & Visual Science*.

[33] Filla M. S., Liu X., Nguyen T. D. (2002). In vitro localization of TIGR/MYOC in trabecular meshwork extracellular matrix and binding to fibronectin. *Investigative Ophthalmology & Visual Science*.

[34] Keller K. E., Bhattacharya S. K., Borrás T. (2018). Consensus recommendations for trabecular meshwork cell isolation, characterization and culture. *Experimental Eye Research*.

[35] Kumar P., Nagarajan A., Uchil P. D. (2018). Analysis of Cell Viability by the MTT Assay. *Cold Spring Harbor Protocols*.

[36] Livak K. J., Schmittgen T. D. (2001). Analysis of relative gene expression data using real-time quantitative PCR and the 2^−*ΔΔ* _C_^_T_ method. *Methods*.

[37] Wu J., Chen S., Liu H. (2018). Tunicamycin specifically aggravates ER stress and overcomes chemoresistance in multidrug-resistant gastric cancer cells by inhibiting N-glycosylation. *Journal of Experimental & Clinical Cancer Research*.

[38] Saccà S. C., Pulliero A., Izzotti A. (2015). The dysfunction of the trabecular meshwork during glaucoma course. *Journal of Cellular Physiology*.

[39] Chakrawarti L., Agrawal R., Dang S., Gupta S., Gabrani R. (2016). Therapeutic effects of EGCG: a patent review. *Expert Opinion on Therapeutic Patents*.

[40] Martinotti S., Ranzato E., Burlando B. (2018). (−)- Epigallocatechin-3-gallate induces GRP78 accumulation in the ER and shifts mesothelioma constitutive UPR into proapoptotic ER stress. *Journal of Cellular Physiology*.

[41] Nesran Z. N. M., Shafie N. H., Ishak A. H., Esa N. M., Ismail A., Tohid S. F. M. (2019). Induction of endoplasmic reticulum stress pathway by green tea epigallocatechin-3-gallate (EGCG) in colorectal cancer cells: activation of PERK/p-eIF2*α*/ATF4 and IRE1*α*. *BioMed Research International*.

[42] Collins Q. F., Liu H. Y., Pi J., Liu Z., Quon M. J., Cao W. (2007). Epigallocatechin-3-gallate (EGCG), a green tea polyphenol, suppresses hepatic gluconeogenesis through 5’-AMP-activated protein kinase. *Journal of Biological Chemistry*.

[43] Elbling L., Herbacek I., Weiss R. M. (2010). Hydrogen peroxide mediates EGCG-induced antioxidant protection in human keratinocytes. *Free Radical Biology & Medicine*.

[44] Zhang Y., Yang N. D., Zhou F. (2012). (-)-Epigallocatechin-3-gallate induces non-apoptotic cell death in human cancer cells via ROS-mediated lysosomal membrane permeabilization. *PLoS One*.

[45] Min N. Y., Kim J. H., Choi J. H. (2012). Selective death of cancer cells by preferential induction of reactive oxygen species in response to (-)-epigallocatechin-3-gallate. *Biochemical and Biophysical Research Communications*.

[46] Nakagawa H., Hasumi K., Woo J. T., Nagai K., Wachi M. (2004). Generation of hydrogen peroxide primarily contributes to the induction of Fe(II)-dependent apoptosis in Jurkat cells by (-)-epigallocatechin gallate. *Carcinogenesis*.

[47] Tang Q., Zheng G., Feng Z. (2017). Trehalose ameliorates oxidative stress-mediated mitochondrial dysfunction and ER stress via selective autophagy stimulation and autophagic flux restoration in osteoarthritis development. *Cell Death & Disease*.

[48] Relat J., Blancafort A., Oliveras G. (2012). Different fatty acid metabolism effects of (-)-epigallocatechin-3-gallate and C75 in adenocarcinoma lung cancer. *BMC Cancer*.

[49] Bhatia N., Agarwal C., Agarwal R. (2001). Differential responses of skin cancer-chemopreventive agents silibinin, quercetin, and epigallocatechin 3-gallate on mitogenic signaling and cell cycle regulators in human epidermoid carcinoma A431 cells. *Nutrition and Cancer*.

[50] Singh T., Katiyar S. K. (2013). Green tea polyphenol, (−)-epigallocatechin-3-gallate, induces toxicity in human skin cancer cells by targeting *β*-catenin signaling. *Toxicology and Applied Pharmacology*.

[51] Wu P. P., Kuo S. C., Huang W. W. (2009). (-)-Epigallocatechin gallate induced apoptosis in human adrenal cancer NCI-H295 cells through caspase-dependent and caspase-independent pathway. *Anticancer Research*.

[52] Kasetti R. B., Patel P. D., Maddineni P. (2020). ATF4 leads to glaucoma by promoting protein synthesis and ER client protein load. *Nature Communications*.

[53] Modernelli A., Naponelli V., Giovanna Troglio M. (2015). EGCG antagonizes Bortezomib cytotoxicity in prostate cancer cells by an autophagic mechanism. *Scientific Reports*.

[54] Tang G. Y., Xu Y., Zhang C., Wang N., Li H., Feng Y. (2021). Green tea and epigallocatechin gallate (EGCG) for the management of nonalcoholic fatty liver diseases (NAFLD): insights into the role of oxidative stress and antioxidant mechanism. *Antioxidants*.

[55] Zhang W. H., Chen Y., Gao L. M., Cao Y. N. (2021). Neuroprotective role of epigallocatechin-3-gallate in acute glaucoma via the nuclear factor‑*κ*B signalling pathway. *Experimental and Therapeutic Medicine*.

[56] Shen C., Chen L., Jiang L., Lai T. Y. Y. (2015). Neuroprotective effect of epigallocatechin-3-gallate in a mouse model of chronic glaucoma. *Neuroscience Letters*.

[57] Wang J., Lee J., Liem D., Ping P. (2017). *HSPA5* Gene encoding Hsp70 chaperone BiP in the endoplasmic reticulum. *Gene*.

[58] McCullough K. D., Martindale J. L., Klotz L. O., Aw T. Y., Holbrook N. J. (2001). Gadd153 sensitizes cells to endoplasmic reticulum stress by down-regulating Bcl2 and perturbing the cellular redox state. *Molecular and Cellular Biology*.

